# Worsening of alcohol abuse disorder in a Spanish population during the first twelve months of the COVID-19 pandemic and associated factors: retrospective, ecological and community study

**DOI:** 10.1186/s12888-023-04993-5

**Published:** 2023-07-12

**Authors:** Bárbara Oliván-Blázquez, Ana Lear-Claveras, Mario Samper-Pardo, Sandra León-Herrera, Rosa Magallón-Botaya

**Affiliations:** 1 Aragonese Research Group in Primary Care (Grupo Aragonés de Investigación en Atención Primaria/GAIAP), Institute for Health Research Aragon (IISAragon), Zaragoza, Spain; 2 Network for Research on Chronicity, Primary Care, and Health Promotion (RICAPPS), Barcelona, 08007 Spain; 3 Department of Psychology and Sociology, University of Zaragoza, Zaragoza, 50.009 Spain; 4 Department of Medicine, University of Zaragoza, Zaragoza, Spain

**Keywords:** COVID-19 pandemic, Alcohol abuse disorder, Worsening, Social care, Health care

## Abstract

**Purpose:**

To analyse: (1) Changes in clinical parameters and in the use of social healthcare resources by patients with alcohol abuse disorder between the six months prior to the start of the pandemic and the first year of the pandemic. (2) The factors related to a worsening of clinical parameters among patients with alcohol abuse disorder.

**Methods:**

A retrospective and observational study of a population who have been diagnosed with alcohol abuse disorders according to their primary health care (PHC) electronic medical records was performed. The total sample was made up of 11,384 patients. The variables (sociodemographic variables, chronic comorbidities, analytical parameters related to alcohol abuse disorder, COVID-19 infection, and use of healthcare resources) were collected in three different time periods: (i) six months before the onset of the strict lockdown, (ii) six months following the end of lockdown and (iii) from six to twelve months after the end of lockdown. Paired Student’s T-test and a multivariate logistic regression were performed.

**Results:**

Along the first year after the onset of the pandemic, between 44% and 54% of the patients suffered a decline in every clinical parameter. The number of PHC nursing, GP visits and social worker visits reduced significantly. As regards the associated factors related to deterioration of alcohol abuse disorder, being younger than 40 years old, having an income of over 18,000 euros/year and not having visited the social worker were associated with a worsening of the disorder.

**Conclusions:**

These results suggest that the impact of COVID-19 on this group has been high, and the social care offered to these patients plays a significant role in minimising the repercussions of the pandemic.

## Introduction

Alcohol dependence and alcohol abuse or harmful use cause substantial morbidity and mortality [1]. It is estimated that there are 237 million men and 46 million women worldwide who suffer from alcohol use disorder. In 2016, the harmful use of this substance caused more than 3 million deaths across the globe (three-quarters of these in men), which accounts for 5% of the global burden of disease [2]. Globally, that same year, alcohol consumption was the seventh highest risk factor for premature death and disability, and was the main risk factor among the population aged between 15 and 49 [3]. Continued heavy alcohol use also accelerates the onset of heart disease, stroke, cancers, and liver cirrhosis, by affecting the cardiovascular, gastrointestinal, and immune systems. So that alcohol use disorders directly and indirectly contribute significantly to the health care cost [4]. Furthermore, the harmful consumption of alcohol not only has physical consequences, but also psychological and social ones [5]. Alcohol use disorders are commonly associated with depression or other mental illnesses (dual pathology), severe anxiety, insomnia, suicide, and the abuse of other drugs [1]. This way alcohol use disorder impairs quality of life damaging interpersonal and social roles (for example: family conflicts, violence or, unemployment) [5].

Alcohol use disorder is associated with important life events and problems. Since 2019, people may have consumed alcohol to reduce the intensity of negative feelings caused by the outbreak of the COVID-19 pandemic [6, 7]. To contain the spread of this new SARS-CoV-2 virus, the governments of many countries imposed restrictive measures that may have been stressful for the population such as restriction of social activities, limited mobility, and/or being locked down at home. Many studies have shown the psychological consequences of the pandemic and lockdown on the mental health of the general population. Several meta-analyses have concluded that the COVID-19 pandemic has exacerbated mental health problems among the global population, mainly the prevalence of depression, anxiety, and sleep disturbances [8–11]. On the other hand, fewer studies have evaluated the effect of this pandemic and the consequent lockdown on alcohol consumption patterns.

In relation to changes in alcohol consumption patterns during the COVID-19 pandemic, several studies and reviews have been carried out, mainly performed on the general population, the results of which diverge significantly [3, 12–16]. In all these studies, psychosocial distress has been recognised as a risk factor for relapse and increased alcohol consumption [17]. However, it is also relevant to analyse the effects of lockdowns and restrictions on alcohol consumption in patients with pre-existing alcohol use disorder. Regarding this last aspect, [17] concluded that lockdowns are a risk factor for increased alcohol consumption among people with alcohol use disorders, and for relapse for those who had previously been abstinent.

In addition, the impact of the COVID-19 crisis on essential healthcare services is of great concern, as healthcare systems and particularly Primary Health Care (PHC) have been focused on treating and detecting cases of the infection, following up positive cases and contact tracing. According to the results of the PULSE survey carried out by the World Health Organization (WHO) in 105 countries, most (90%) have suffered interruptions in essential healthcare services since the beginning of the pandemic [18]. These interruptions are likely to have serious adverse effects on the health of the most vulnerable populations, such as those with chronic diseases who need regular assistance and care, especially in PHC settings [19].

To the best of our knowledge, few studies have been performed which analyse the effect of the COVID-19 pandemic that consider not only the effects of the lockdown but also the health, social, political, and economic crisis, looking specifically at people with a history of alcohol abuse disorder, and using a longitudinal, ecological and community methodology. The use of data from PHC records allows us to quantify changes in clinical parameters, as well as the consumption of healthcare resources, with such changes possibly indicating a modification in the state of the illness. Primary healthcare is the gateway to the health system, and the closest community healthcare resource to the population. Furthermore, epidemiological analyses using real world data (RWD) such as data from primary healthcare records provide results related to decision-making from the perspective of health and social care.

Hence, the aims of this study are to analyse changes in clinical parameters and in the use of social healthcare resources by patients with alcohol abuse disorder between the six months prior to the start of lockdown and the twelve months after it was lifted. We will be focusing on primary healthcare patients diagnosed with alcohol abuse disorder in one of Spain’s 17 ‘autonomous communities’, Aragon, situated in the north of the country. We also aim to analyse the factors related to a worsening of clinical parameters among these patients.

## Methodology

### Study design

This is a retrospective, ecological and observational study of a population in northern Spain (Aragon) over the age of 16 who have been diagnosed with alcohol abuse disorders according to their primary health care electronic medical records (EMR), using RWD. This massive complex and varied data set led to the use of technological tools to be processed. The clinical data management system of the Aragonese Health Service (BIGAN) collected all the data generated by the health system and anonymized them. This big data tool made it possible to build a database that included sociodemographic and clinical information of those patients older than 16 years with EMR in Primary Care and with a diagnosis of alcohol abuse disorder at least 6 months before the declaration of the state of alarm in Spain.

### Sample and sample size

The sample consisted of all patients with an open EMR held by health centres in the Autonomous Community of Aragon (Spain) with a diagnosis of alcohol abuse disorder at least 6 months before the declaration of the state of alarm in Spain (14/09/2019). Accordingly, the inclusion criteria were: (i) over 16 years old, (ii) registered diagnosis of alcohol abuse disorder (code F10 in the 10th revision of the International Statistical Classification of Diseases and Related Health Problems (ICD-10)). The exclusion criterion was to minimise inconsistencies in the data.

The total sample that met these criteria was made up of 11,384 patients. Due to the universal nature of the healthcare system and the absence of other PHC providers, the data obtained in the study is considered representative of practically 100% of the studied population.

### Study variables

Data on sociodemographic variables, chronic comorbidities, analytical parameters, COVID-19 infection, and use of healthcare resources were collected from PHC records for the three distinct periods. The first measurement was taken from the records from the 6 months before the onset of the pandemic, which began with a strict lockdown (14/09/2019 to 14/03/2020). The second period covers the 6 months following the end of this lockdown during the first wave (03/05/2020 to 04/11/2020), and the third measurement was taken from the records for months 6–12 after the end of lockdown (05/11/2020 to 05/05/2021). The measurements taken during lockdown have not been included because there is an extremely small number of assessments and therefore there was not enough statistical power.

- Sociodemographic variables: sex, age, individual´s income collected from prescription charge records (categorised by those earning less than 18,000 euros/year, 18,000 to 100,000 euros/year, over 100,000 euros/year, and those who are uninsured and receive free prescriptions), social service assistance at primary health centres, and residence in a rural or urban area (urban is defined as an area with a population of over 10,000).
- Data on chronic comorbidities presenting a prevalence of over 5% in the general population [20] were collected at six months before the lockdown. New diagnosis during the six and twelve months after the end of lockdown were also registered. The chronic pathologies included were: (i) physical: arrhythmias, heart failure, ischemic heart disease, hypertension, dyslipidaemia, obesity, excess weight, vein and artery disease, cerebrovascular disease, diabetes, chronic bronchitis, chronic obstructive pulmonary disease (COPD), asthma, chronic kidney disease, hypo- and hyperthyroidism, anaemia, neoplasia, hearing loss, cataracts, glaucoma, osteoarthritis, osteoporosis, back pain, and (ii) psychiatric: nicotine addiction, depression and anxiety, insomnia, autolytic attempt, and dementia.
- Analytical parameters related to chronic alcohol consumption were included: glutamic pyruvic transaminase (GPT), glutamic oxaloacetic transaminase (GOT), blood creatinine, and glomerular filtration. An increase in these parameters and a decrease in the last one indicate a deterioration of the illness. The main variables that indicate deterioration are GPT and GOT, which reflect decreased liver function due to alcohol consumption. The normal values for GPT are between 8 and 35 IU/L for males, and between 6 and 25 IU/L for females. The values for GOT used as a normal reference point are between 8 and 30 IU/L for men, and between 6 and 25 IU/L for women. Normal blood creatinine levels are between 0.7 and 1.3 mg/dL for men, and between 0.6 and 1.1 mg/dL for women. Finally, glomerular filtration has been calculated taking age and sex into account. Values lower than 60 mL/min/1.73m^2^ are indicative of kidney malfunction. In cases where an increase in these parameters or a decrease in glomerular filtration was found (comparing the basal level and that at 6 or 12 months post-lockdown), it was considered that the disease had deteriorated.
- Infection with COVID-19 during the study period, classified as yes/no.
- Use of healthcare resources by these patients was assessed by looking at use of PHC services. This included the number of ordinary or continuous care visits to the health centre or home visits by the nurse or General practitioner (GP), the number of visits to other health professionals at the health centre (social worker, odontologist, physical therapist), and the use of hospital services (number of specialised care visits, number of visits to accident and emergency [A&E] services, hospitalisations, and admission to intensive care units [ICU], and the duration of these stays) for each of the periods under study.

### Statistical analysis

The sample size allowed the use of parametric methods [21]. Firstly, a descriptive analysis of the study variables was carried out using frequencies, means and standard deviation (SD) according to the nature of the variable (dichotomy or continuous variables respectively).

To determine variations in the analytical parameters, the differences between the GPT, GOT, blood creatinine and glomerular filtration levels for each period (6 months prior to lockdown, 6 months and 12 months after lockdown was lifted) were calculated. Patients with data before the outbreak of the pandemic and 6 months and/or 12 months after were included. To compare the differences in the use of healthcare resources for each period, a paired Student’s T-test was used. For those variables with a frequency of less than 100, a Wilcoxon rank test was used.

To analyse associated factors related to the possible worsening of the illness, a multivariate logistic regression was performed. Worsening of the illness was considered to have taken place when GPT and/or GOT values increased, which was analysed as dependent variable. The independent variables were sex; age (categorised as younger than 40 years old, from 40 to 60 years old, and older than 60 years old); income according to prescription charge bracket (income of less or more than 18,000 euros/year); social assistance (having visited the PHC social worker; not having visited the PHC social worker); residence in a rural or urban area; chronic comorbidities (categorised as not presenting comorbidity or presenting another chronic disease); and COVID-19 infection.

Statistical analysis was carried out using IBM SPSS Statistic 21 [22] and R 4.0.5 [23]. on a PC with 16 MB of RAM.

### Ethical considerations

The Study Protocol was approved by the Aragon Clinical Research Ethics Committee (PI20-175). All procedures contributing to this work comply with the ethical standards of the Aragon Clinical Research Ethics Committee (part of the Department of Health of the Government of Aragon) and with the Helsinki Declaration of 1975, as revised in 2008. Data were obtained from clinical records provided in a non-identifiable format by the Aragon Health Service. The processing, notification, and transfer of personal data were carried out in accordance with Regulation (EU) 2016/679 of the European Parliament and Spanish Organic Law 03/2018 on the Protection of Personal Data and the guarantee of digital rights.

## Results

In Aragon, on 14/09/2019 there were 11,384 patients diagnosed with alcohol abuse disorder by their GP, with males accounting for 84.1% of the total. These patients’ mean age is 56.04 (13.05), 71.5% of them had an annual income of less than 18,000 euros, and 8.1% had been infected with COVID-19. In terms of the presence of comorbidities, dyslipidaemia (45.3%), nicotine addiction (44.1%), hypertension (35.5%), depression and anxiety (35.5%), back pain (23.7%) and neoplasia (22.2%) were the most frequent chronic conditions in the study population. The analytical parameters that can indicate liver damage were found to show normal levels in both males (GPT: 29.62 SD: 17.29; GOT: 30.48 SD:15.96) and females (GPT: 20.43 SD:1.55; GOT:22.70 SD:9.75) before the pandemic outbreak. Other analytical parameters such as blood creatinine and glomerular filtration also showed normal levels. Table 1 describes the sample according to the studied variables.

**Table 1:** Sociodemographic data, chronic comorbidities, clinical parameters, and COVID-19 infection in patients suffering from alcohol abuse disorder at 6 months before the pandemic outbreak

		N = 11,384
Sex		
	Male (%) Female (%)	9576 (84.1) 1808 (15.9)
Age, *M (SD)*		56.04 (13.05)
Prescription charge brackets		
	< 18,000, *n (%)*	8137 (71.5)
	Between 18,000–100,000, *n (%)*	2285 (20.1)
	> 100,000, *n (%)*	19 (0.2)
	Free prescriptions, *n (%)*	927 (8.1)
	Uninsured, *n (%)*	6 (0.1)
Residence		
	Urban, *n (%)*	6321 (55.5)
	Rural, *n (%)*	5063 (44.5)
COVID-19 infection, *yes n (%)*		922 (8.1)
Chronic comorbidities (Physical)		
	Arrhythmias, *yes n (%)*	574 (5.0)
	Heart failure, *yes n (%)*	227 (2.0)
	Ischemic heart disease, *yes n (%)*	570 (5.0)
	Hypertension, *yes n (%)*	4040 (35.5)
	Dyslipidaemia, *yes n (%)*	5161 (45.3)
	Obesity, *yes n (%)*	1524 (13.4)
	Excess weight, *yes n (%)*	205 (1.8)
	Vein/artery disease, *yes n (%)*	564 (5.0)
	Cerebrovascular disease, *yes n (%)*	524 (4.6)
	Diabetes, *yes n (%)*	1751 (15.3)
	Chronic bronchitis, *yes n (%)*	216 (1.9)
	COPD, *yes n (%)*	1121 (9.8)
	Asthma, *yes n (%)*	518 (4.6)
	Chronic kidney disease, *yes n (%)*	394 (3.5)
	Hypothyroidism, *yes n (%)*	580 (5.1)
	Hyperthyroidism, *yes n (%)*	196 (1.7)
	Anaemia, *yes n (%)*	1312 (11.5)
	Neoplasia, *yes n (%)*	2527 (22.2)
	Hearing loss, *yes n (%)*	878 (7.7)
	Cataracts, *yes n (%)*	835 (7.3)
	Glaucoma, *yes n (%)*	483 (4.3)
	Osteoarthritis, *yes n (%)*	563 (4.9)
	Osteoporosis, *yes n (%)*	288 (2.5)
	Back pain, *yes n (%)*	2695 (23.7)
Chronic comorbidities (Psychiatric)		
	Nicotine addiction, *yes n (%)*	5019 (44.1)
	Depression and anxiety, *yes n (%)*	4044 (35.5)
	Insomnia, *yes n (%)*	1712 (15.0)
	Autolytic attempt, *yes n (%)*	248 (2.2)
	Dementia, *yes n (%)*	187 (1.6)
Analytical parameters		
	GPT	
	Men *mean (SD)* Women *mean (SD)*	29.62 (17.29) 20.43 (11.55)
	GOT	
	Men *mean (SD)* Women *mean (SD)*	30.48 (15.96) 22.70 (9.75)
	Blood creatinine *mean (SD)*	
	Men *mean (SD)* Women *mean (SD)*	0.89 (0.23) 0.74 (0.29)
	Glomerular filtration *mean (SD)*	90.38 (18.67)

*Note*. COPD, Chronic obstructive pulmonary disease.

As for new diagnoses of psychiatric comorbidity in this population, in the 6 months before the lockdown, there were 115 (1%) new diagnoses of nicotine addiction, 186 (1.6%) of depression and anxiety, 92 (0.8%) of insomnia and 15 (0.1%) attempted suicides. However, in the 6-month period after lockdown and the 6 months thereafter, there were, respectively, 31 (0.3%) and 11 (0.1%) new diagnoses of nicotine addiction, 143 (1.3%) and 34 (0.3%) of depression and anxiety, 69 (0.6%) and 19 (0.2%) of insomnia, and 20 (0.2%) and 2 (0.01%) suicide attempts. As can be observed, there is a decrease in the incidence of psychiatric pathology.

The number and percentage of patients with alcohol abuse disorder and their clinical parameters (GPT, GOT, blood creatinine and glomerular filtration) at 6 months and 12 months post-lockdown are shown in Table 2. As can be observed, between 44% and 54% of the patients suffered a decline in every clinical parameter (increase in GPT, GOT and blood creatinine and decrease in glomerular filtration), both at 6 months post-lockdown and at 12 months post-lockdown. Approximately 50% of people with alcoholism worsen in the parameters related to this pathology at each time point.

**Table 2:** Number and percentage of alcohol abuse disorder patients by clinical parameter (GPT, GOT, blood creatinine and glomerular filtration) with a stable, improvement or worsening level at 6 months and 12 months post-lockdown

Clinical parameter	N	Stable or improvement at 6 months	Worsening at 6 months	N	Stable or improvement from months 6 to 12	Worsening from months 6 to 12
GPT	112	57 (50.89)	55 (49.11)	156	87 (55.77)	69 (44.23)
GOT	102	48 (47.05)	54 (51.95)	140	73 (52.14)	67 (47.86)
Blood creatinine	132	70 (53.03)	62 (46.97)	176	85 (48.29)	91 (51.71)
Glomerular filtration	132	66 (50.00)	66 (50.00)	176	96 (54.54)	80 (45.46)

As is seen in Table 3, the number of PHC nursing and GP visits, ordinary and/or continuous, at the healthcare centre and/or home visits, reduced significantly during the first six months post-lockdown, but also from 6 to 12 months post-lockdown. The number of social worker visits decreased significantly the first 6 months post-lockdown. Visits to hospital services (no. of visits to A&E services and no. of hospitalisations) also decreased significantly during the study period. On the other hand, the number of visits to specialised care increased significantly during the period from 6 to 12 months post-lockdown. On the other hand, it should be noted that the number of people who needed or requested home care, both nursing and family medicine, comparing the period prior to the pandemic, the 6 months after the onset and from 6 to 12 months, increased significantly in the last period of time collected (6 to 12 months).

**Table 3:** Number (Mean and SD) of visits to PHC professionals at six months pre-lockdown, six months post-lockdown, and 12 months post-lockdown

	N	Six months pre-lockdown	Six months post-lockdown	(95% CI) P-value	N	Six months pre-lockdown	12 months post- lockdown	(95% CI) p value
No. of nursing visits (ordinary care) at health centre or by telephone	3246	4.65 (6.47)	4.28 (5.97)	(0.15;0.58) 0.001	2889	4.64 (6.31)	4.04 (5.13)	(0.36;0.83) < 0.001
No. of nursing home visits (ordinary care)	167	6.07 (9.58)	6.34 (12.91)	(-1.68;1.13) 0.700	257	4.06 (7.54)	2.93 (7.65)	(0.26;2.01) 0.110
No. of nursing visits (continuous care) at health centre	332	2.53 (3.31)	1.98 (1.97)	(0.21;0.89) 0.001	333	2.07 (3.06)	2.61 (6.35)	(-0.23;0.59) 0.403
No. of nursing home visits (continuous care)	44	2.61 (6.35)	2.29 (5.73)	(0-0.18;0.81) 0.135^a^	64	2.01 (5.33)	1.07 (3.50)	(0.28;1.58) < 0.001 ^a^
No. of GP visits (ordinary care) at health centre or by telephone	7121	5.35 (5.11)	5.77 (5.80)	(-0.55; -0.27) < 0.001	6871	5.28 (5.05)	5.13 (4.89)	(0.01;0.29) 0.041
No. of GP home visits (ordinary care)	106	2.59 (2.30)	1.72 (3.49)	(0-0.21; 0.92) 0.205	391	1.72 (1.49)	0.71 (1.64)	(0.83;1.17) < 0.001
No. of GP visits (continuous care) at health centre	699	2.01 (1.84)	2.14 (2.44)	(-0.31; 0.05) 0.166	602	2.02 (1.95)	1.89 (1.96)	(-,05;0.31) 0.160
No. of GP home visits (continuous care)	55	1.61 (0.84)	1.36 (0.91)	(-0.08; 0.59) 0.082 ^a^	101	1.34 (0.92)	0.51 (0.72)	(0.60;1.06) < 0.001 ^a^
No. of visits to PHC social worker	74	2.70 (2.66)	4.18 (4.35)	(-2.56; -0.40) 0.009 ^a^	78	2.55 (2.53)	2.58 (3.02)	(-0.74; 0.66) 0.730 ^a^
No. of visits to specialised care	2215	2.74 (2.38)	2.72 (2.71)	(0.16; 0.39) 0.735	2161	2.06 (2.43)	2.37 (2.46)	(0.16; 0.39) < 0.001
No. of visits to A&E department	790	2.12 (2.37)	2.05 (2.45)	(-,09; 0.23) 0.442	696	2.06 (2.43)	1.86 (1.75)	(0.01; 0.38) 0.033
No. of hospital admissions	796	1.35 (0.77)	0.39 (0.83)	(0.88; 1.03) < 0.001	166	1.42 (0.88)	1.65 (1.23)	(-0.43; -0.02) 0.027
No. of ICU admissions	4			*	1			*

*Note*. CI: Confidence Interval; PHC: Primary Health Care; A&E department: Accident and Emergency department; ICU: Intensive Care Unit. ^a^ Wilcoxon signed - rank test. * The correlation and t cannot be calculated because the standard error of the difference is 0

**Fig. 1:**
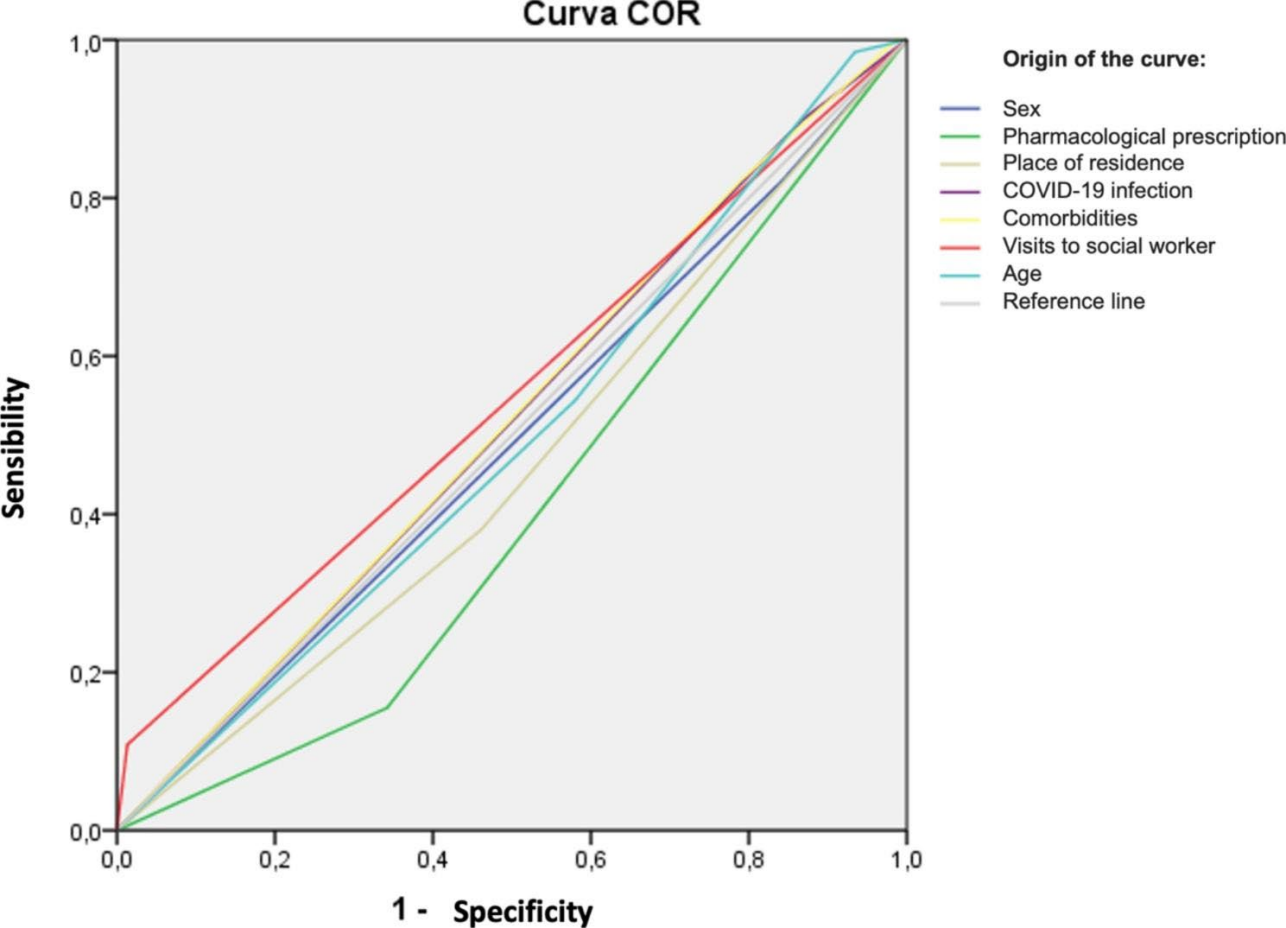
ROC curve of the logistic regression model

As regards the associated factors related to deterioration of alcohol abuse disorder, to examine the patients whose GPT and/or GOT levels worsened at 6 months or 12 months post-lockdown, a multivariable logistic regression was performed, the results of which are displayed in Table 4. A significant model was obtained (p-value = 0.001), with a Cox and Snell R-squared of 0.108. Having an income of over 18,000 euros/year (p-value 0,003) and not having visited the social worker (p-value 0,022) are associated with a deterioration in the clinical parameters that would indicate a worsening of alcohol abuse disorder during the first year of the COVID-19 pandemic. Being younger than 40 years old was also found to be significant in this sense (p-value 0,075). Figure 1 shows the ROC curve of the logistic regression model.

**Table 4:** Multivariable logistic regression of factors associated to a deterioration of alcohol abuse disorder, indicated by an increase in GPT and/or GOT values at 6 months or 12 months post-lockdown

		B	Exp (B) Odds ratio	95% Confidence Interval for Exp(B)	P value
Intercept		-2.014			0.073
Sex					
	Male	0^b^	Ref		
	Female	0.064	1.066	0.470; 2.419	0.879
Age					
	Over 60 years	0^b^	Ref		
	40 to 60 years	-0.144	0.865	0.457; 1.639	0.658
	Under 40 years	1.768	5.859	0.834; 41.147	0.075
Prescription charge brackets					
	Income < 18,000 euros/year	0^b^	Ref		.
	Income > 18,000 euros/year	-1.097	0.334	0.163; 0.686	0.003
Social assistance					
	Visit to PHC social worker	0^b^	Ref		.
	No visit to PHC social worker	2.484	11.994	1.432; 100.469	0.022
Residence					
	Residence in rural area	0^b^	Ref		.
	Residence in urban area	-0.200	0.819	0.439; 1.527	0.530
Chronic comorbidities					
	Any comorbidity	0^b^	Ref		
	No comorbidity	1.050	1.050	0.253; 4.360	0.947
COVID-19 infection					
	No COVID-19 infection	0^b^	Ref		.
	COVID − 19 infection	0.325	1.384	0.531; 3.604	0.506

## Discussion

This study analyses the evolution of a sample consisting of patients who had been diagnosed with alcohol abuse disorder before the COVID-19 pandemic, at least six months before lockdown. Exposure to other serious infectious diseases (SARS) has already been associated with alcohol abuse/dependence [24]. Nevertheless, SARS outbreak had less global impact than SARS-CoV-2 and the measures implemented were less restrictive. COVID-19 pandemic is an exceptional and unprecedented situation so its effects on alcohol abuse/dependence can be expected to be greater [25]. The stressful situation experienced by the population and the saturation of the healthcare system could have caused a deterioration of the disease. The results of our study, considering that an increase in the analytical parameters indicate a worsening of the illness, show that around the 50% of patients saw a deterioration in these biological markers. This could be indicative of a higher consumption of alcohol during the period under study.

Some studies show results which indicate that the population with alcohol use disorder increased their alcohol consumption during the health crisis [17, 26–28], as well as an increase in the number of relapses during the months of lockdown. Those who experienced emotional distress or lived alone at the onset of the pandemic had a higher risk of quitting abstinence. On the other hand, other studies show stability [29] or even a decrease in alcohol consumption [30, 31] during the COVID-19 pandemic.

This disparity in results may be explained by the data resources used, but also by the periods under study and the care provided during the COVID-19 pandemic. Some studies [17, 29, 31] collected data through self-reported questionnaires without evaluating biological markers. Barrio et al.’s study [26], which uses biological markers and was also carried out in Spain, shows results similar to our own.

The point of data collection may be also relevant when explaining the results, since some studies analysed data during lockdown, and immediately or 6 months after it was lifted [26, 29]. Our study looks at the 12 months post-lockdown. The COVID-19 pandemic has lasted for over two years, so it is necessary to analyse the repercussions on the population’s health, not only in the short term but also in the long term.

Fear of infection and the collapse of the healthcare system, especially primary care services, during every wave of the pandemic has interrupted continuous healthcare services for all patients [18, 32, 33]. This may have caused psychopathological imbalances. In our study, a general decrease in the number of visits to primary health care centres by patients with alcohol abuse disorder is observed, compared to the six months pre-lockdown.

To overcome these interruptions, telemedicine was established as the most frequently used option to replace in-person appointments and to offer psychosocial support to patients [33, 34]. Telemedicine was consolidated as one of the most frequently used and effective alternatives to overcome these interruptions [35, 36], and could explain the increase in the number of ordinary face-to-face or telephone GP visits observed in our study during the first 6 months of the pandemic. Virtual or face-to-face consultations during the pandemic have been shown to be instrumental in reducing relapses (p = 0.075; OR 0.303, 95% CI 0.110–0.839) and helping people to begin abstaining (p = 0.035; OR 1.118, 95% CI 0.032–0.432), compared with those who did not have contact with a health professional [17].

The increase in demand for social services in the first months after the pandemic is also significant, reflected by the notable increase in the number of consultations with social workers. This data, according to Druss´ study [37], is consistent with the social and economic impact of COVID-19 and evinces the devastating psychological distress vulnerable people have suffered from. The relationship between mental health and economic crisis is well established in the literature [38–41].

Looking at the long term, it is worth highlighting the increase in the number of visits to specialised care and hospital admissions at 12 months post-lockdown. This may indicate a physical and psychological imbalance in these patients’ health. This idea is supported by most of the existing literature [8, 10, 11, 29, 37, 42–44]. However, there have been fewer new diagnoses (especially psychiatric diagnoses) among these patients by their GPs, which could indicate an underdiagnosis in primary health care.

Factors associated with a worsening of clinical parameters include having an income of over 18,000 euros/year and not having visited the social worker at the health centre. In relation to patient’s socio-economic situation, several studies have evaluated the cross-sectional relationship between socio-economic status and alcohol abuse, concluding that medium and high incomes were correlated with a greater frequency of alcohol abuse, higher than among those with a lower income [45, 46]. In addition, there is a higher prevalence among young adults whose families have greater economic wealth [47]. Likewise, more affluent neighbourhoods present a higher prevalence of alcohol consumption, especially if there is a great disparity in income, causing greater consumption among lower socio-economic groups [45, 48–50]. Even so, it is necessary to view these data with caution, given that several studies contemplate other social determinants that can alter said evidence for the male sex, such as socio-economic status and employment status, with a relationship having been identified between educational poverty and unemployment with higher alcohol intake and consequently high mortality rates related to substance abuse [51–55]. High income is associated with ‘hazardous use’ of alcohol, a DSM-V substance abuse criterion, such as driving after consuming alcohol [56].

On the other hand, in relation to visits to a health centre social worker, during the first year of the pandemic various studies indicate that there has been a negative impact on the quality of life of those sick with COVID-19 and of the general population, at a physical, mental, social and economic level [57, 58], as well as an increase in discomfort in the family home [59–61]. All this data has been used to promote social work referrals, which has facilitated the consideration of different treatment options for various health problems such as alcohol abuse. Social work professionals have practical tools to promote prevention and complement psychosocial rehabilitation treatment for addiction to alcohol and other drugs, which can alleviate the effects of withdrawal and various problems that may contribute to a relapse [62–65]. This, in turn, contributes to avoiding a worsening of clinical parameters among affected patients.

The results of our study may help improve the prevention and health care of people with alcohol use disorder. The factors related to a worsening of this disease make it possible to know the most vulnerable population. In this way, preventive strategies must address different population groups and, especially, the one obtained in this study: being under 40 years of age, having an income of more than 18,000 euros/year and not having visited the social worker. These results, added to the increase in demand after the pandemic, make it necessary to strengthen the specialized care network of the health system, offering greater coverage. Special mention for social work professionals as a key player in the care of people with alcohol abuse disorder.

Our study has some significant strengths. Its main strength is the availability of a large population database and the use of clinical-administrative data from the electronic health records of Primary Care. The evidence generated provides greater knowledge about the consequences of the pandemic in vulnerable populations such as patients with alcohol use disorder. On the other hand, it has limitations. The first limitation is that we have considered only data from electronic medical records in three time periods, what prevented knowing changes in clinical parameters during all the twelve months. The second limitation is that we do not have access to a quantified record of alcohol consumption in standard drinking units, nor to some of the specific structured questionnaires used in PC such as the CAGE or the Alcohol Use Disorders Identification Test (AUDIT). The third limitation is that we have considered an increase in the analytical parameters as an indication of deterioration, without determining whether these increases were significant. If we compare clinical parameters before and after the pandemic, these increases may not be significant [63], but may simply indicate a trend. Lastly, we do not have self-reported data about the lifestyles maintained by patients during these months and neither about the current status of the patients (detoxification, cessation or rehabilitation) therefore our results should be interpreted with caution.

It would be interesting to carry out studies with a qualitative approach. That would give us more information about the perceptions that these patients have about the impact of COVID-19 on their health. Also, the use of geographic information systems would allow us to represent through maps the most affected basic health areas identifying geographic clusters.

## Conclusions

Our study sheds some light on the consequences of the COVID-19 pandemic in a large sample of people diagnosed with alcohol abuse disorder from a longitudinal, ecological and community perspective. The results suggest that the impact of COVID-19 on this group has been high, and the social care offered to this group of patients plays a significant role in minimising the repercussions of the pandemic.

## Data Availability

Requests for any underlying data cannot be granted by the authors because the data was acquired under a license/data sharing agreement with the Aragon Health Services, under which conditions of use (and further use) apply. Every request will be studied. Requests to access these datasets should be directed to MS-P (msamperpardo@gmail.com).
